# The Cell Wall Integrity Signaling Pathway and Its Involvement in Secondary Metabolite Production

**DOI:** 10.3390/jof3040068

**Published:** 2017-12-06

**Authors:** Vito Valiante

**Affiliations:** 1 Leibniz Research Group Biobricks of Microbial Natural Product Syntheses, Leibniz Institute for Natural Product Research and Infection Biology-Hans Knöll Institute (HKI), Beutenberg Strasse 11a, 07745 Jena, Germany; vito.valiante@leibniz-hki.de; Tel.: +49-3641-532-1090; 2 Department of General Microbiology and Microbial Genetics, Institute of Microbiology, Faculty of Biology and Pharmacy, Friedrich Schiller University Jena, Neugasse 24, 07743 Jena, Germany

**Keywords:** mitogen-activated protein kinase 1 (Mpk1), mitogen-activated protein kinases (MAPKs), cell wall integrity pathway, melanin, secondary metabolites

## Abstract

The fungal cell wall is the external and first layer that fungi use to interact with the environment. Every stress signal, before being translated into an appropriate stress response, needs to overtake this layer. Many signaling pathways are involved in translating stress signals, but the cell wall integrity (CWI) signaling pathway is the one responsible for the maintenance and biosynthesis of the fungal cell wall. In fungi, the CWI signal is composed of a mitogen-activated protein kinase (MAPK) module. After the start of the phosphorylation cascade, the CWI signal induces the expression of cell-wall-related genes. However, the function of the CWI signal is not merely the activation of cell wall biosynthesis, but also the regulation of expression and production of specific molecules that are used by fungi to better compete in the environment. These molecules are normally defined as secondary metabolites or natural products. This review is focused on secondary metabolites affected by the CWI signal pathway with a special focus on relevant natural products such as melanins, mycotoxins, and antibacterial compounds.

## 1. Fungal Secondary Metabolites

Fungi are organisms able to colonize every habitat across the world. Like plants, fungi do not actively move, and they spread in the environment by producing small spores that can be easily transported by atmospheric carriers (e.g., wind and water flows) and animals. As with every organism on earth, during their life cycle, fungi are actively challenged by environmental changes, e.g., parasites and predators, and they have evolved an incredible ability to adapt to different ecological niches and compete with other organisms. In particular, fungi can produce a large arsenal of compounds that are specifically synthetized to increase their fitness. Many of these active molecules are produced in secondary metabolism and include a wide range of low-molecular-weight chemicals [1]. These chemicals are normally classified as natural products (NPs) and they belong to the class of polyketides, small peptides, terpenoids, alkaloids, and phenols [1]. The different roles of NPs are vast and they are likely produced not only to serve as defense compounds, but also as toxins, which can be relevant for infection processes. Additionally, some NPs can act as metal chelators, and are produced to reduce the fitness of competing organisms by subtracting relevant nutrients, making them more available for the NP-producing organism [2].

Fungal NPs have been employed for diverse applications including antibiotics, antifungals, insecticides, food preservatives, and so on, demonstrating how they are becoming extremely significant for humankind. For this reason, NPs have been largely studied in the last century regarding their synthesis and production. As an example, we know that many fungal NP biosynthetic genes are clustered in their respective genomes and usually entail large multidomain enzymes such as polyketide synthases (PKSs) and/or nonribosomal peptide synthetases (NRPSs) [1,3].

A lot of effort has also been spent on understanding the regulation of NP biosynthesis. In particular, these efforts were focused not only on promoting the increase of NP production, relevant for industrial applications, but also on inducing the expression of silent biosynthesis gene clusters which can potentially disclose new molecules with valuable functions [4]. Specifically, the induction of silent gene clusters became very popular in the post-genomic era. The large and increasing availability of genome data revealed that the knowledge about NPs is very limited if compared with that concerning the computationally identified biosynthetic pathways. This is true even for those organisms that have been extensively studied, which do not produce all their potential chemicals during lab fermenting conditions.

## 2. Translating Stress to Stress Response

Fungal cells are characterized by the presence of a rigid external layer denominated as the cell wall. The fungal cell wall is a dynamic structure that changes during cellular growth, but, during these changes, still conserves its rigidity. The fungal cell wall is principally composed of sugars, such as glucans and chitins, and cell-wall-associated proteins. In many fungi, important components of the cell wall are melanins, which are among the most mysterious secondary metabolites studied. Many genes coding for melanin biosynthesis are well characterized, but the chemical structures of the many different identified melanins are still missing [5,6]. Our knowledge is mainly based on melanin precursors that have been isolated, but the last biosynthetic steps are, so far, unknown [5,6].

The fungal cell wall is also the first layer that interacts with the environment. All the external stimuli need to bypass the cell wall; these signals are then translated to specific responses. Consequently, cell wall stress is potentially responsible for the induction of a series of stress-responsive factors, including the production of secondary metabolites. After crossing the cell wall, stimuli are translated through complex cellular signals, which normally engage phosphorylation cascades.

Signaling pathways are very well conserved in fungi. The most studied signaling pathways in fungi are: the cAMP pathway, which exploits the formation of cyclic AMP to amplify the activity of responsive kinases; the calcineurin pathway, which responds to intracellular calcium homeostasis; the TOR pathway (target of rapamycin), which mainly regulates cellular nutrient and energy levels; and the mitogen-activated protein kinase (MAPK) signaling pathways [7,8,9]. The cell wall integrity (CWI) signaling is one of the MAPK signaling pathways. MAPK pathways are highly conserved among eukaryotes, and are distinguished by a central module composed of three protein kinases. All fungi usually contain three main MAPK pathways: the pheromone responsive pathway, the high osmolarity glycerol (Hog) response pathway, and the CWI pathway. These pathways are strongly connected to each other, and extensive cross-talk interactions are emerging [10,11,12].

## 3. The Cell Wall Integrity Pathway Affects the Production of Melanins

**Figure 1:**
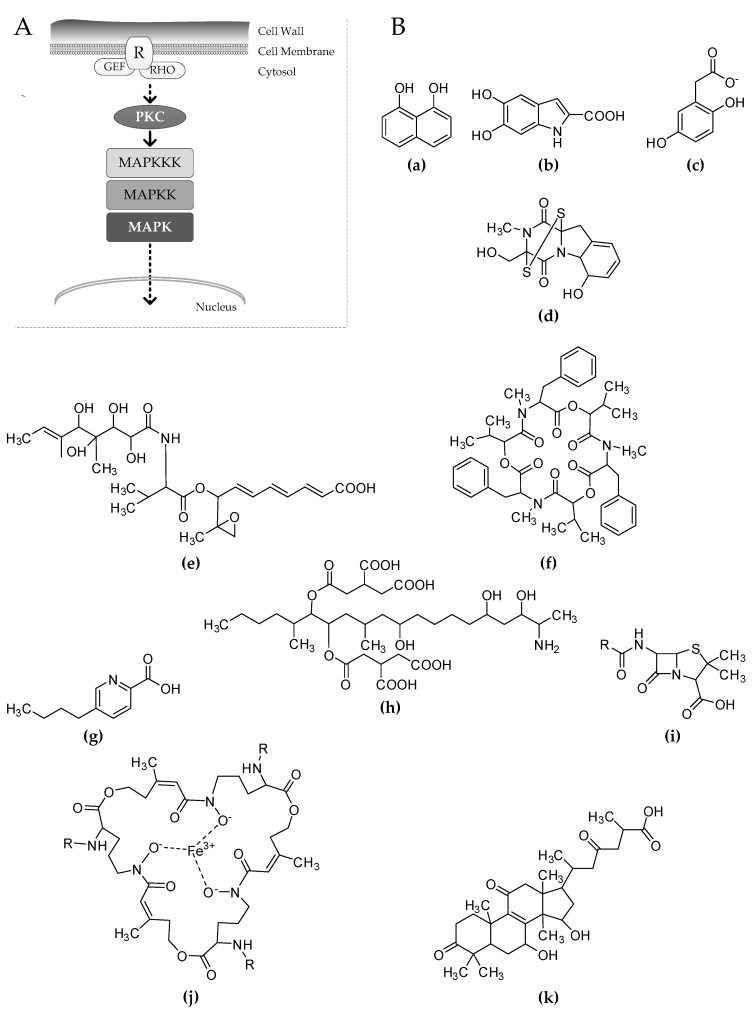
(**A**) Schematic representation of the cell wall integrity (CWI) signaling pathway in fungi. Stress signals from the cell wall are sensed by receptors (R), which are strongly associated with GTPases such as guanidine nucleotide exchange factors (GEFs) and Ras homologs (RHOs). The phosphorylation cascade begins through the protein kinase C (PKC) that activates by phosphorylation of the MAPK module. After being phosphorylated, the MAPK moves to the nucleus, promoting gene transcription through the activation of specific transcriptional regulators. (**B**) Secondary metabolites differentially regulated consequent to the inhibition of the CWI signaling. 1,8-dihydroxynaphthalene (**a**), homogentisate (**b**), and dihydroxyindol (**c**) are known precursors of 1,8-dihydroxynaphthalene (DHN)-melanin, l-Dopa melanin, and pyomelanin respectively. Other represented molecules are: gliotoxin (**d**), ACT toxin (**e**), beauvericin (**f**), fusaric acid (**g**), fumonisin B1 (**h**), penicillin (**i**), triacetylfusarinine C (**j**), and ganoderic acid (**k**).

The CWI pathway was extensively studied in the model fungus *Saccharomyces cerevisiae*, but computational analysis and experimental data revealed that this pathway is highly conserved in fungi (Figure 1A) [8]. The phosphorylation cascade in the MAPK CWI pathway goes from a MAP kinase kinase kinase (MAPKKK), which phosphorylates a MAP kinase kinase (MAPKK) that, at the end, activates a MAPK (Figure 1A). After being phosphorylated, the MAPK moves to the nucleus to activate transcriptional regulators. The majority of fungi contain one element for each kinase, but there are few exceptions reported—in particular, among yeasts [8]. The phosphorylation of the three-kinase module occurs through an additional kinase, a protein kinase C named as Pkc1 in yeast, which is also much conserved. The lack of function of any Pkc1 orthologue results in a lethal phenotype, suggesting that this kinase constitutes a hub for different signaling pathways. Oppositely, the lack of function of any of the three kinases composing the MAPK module, when no paralogues are present, only blocks the phosphorylation cascade and constitutively silences the CWI pathway.

In the model yeast *S. cerevisiae*, the central role in the fungal CWI is played by the MAPK Mpk1 (alias Slt2) [13]. This protein is activated by phosphorylation from two other MAPKKs, named Mkk1 and Mkk2, which are also phosphorylated by the upstream kinase Bck1 (MAPKKK) [14]. The lack of Mpk1 phosphorylation reveals a characteristic phenotype, with compact and delayed growth and high sensitivity to a variety of cell-wall-acting compounds [14]. So far, the deletion of any *mpk1* orthologue in fungi has revealed phenotypes similar to the one reported in yeast, highlighting the conserved role of the CWI pathway. Additionally, the severe physiological alterations due to CWI inhibition strongly affect the virulence in both plant- and human-pathogenic fungi [8,15].

As already mentioned, many fungi produce melanins that are secondary metabolites strongly associated with the cell wall. Melanins are dark pigments synthetized to defend organisms from external hazards [6]. In particular, they play an important role in the defense against reactive oxygen species and UV stress, but they are also important for pathogenesis [6,16]. The chemical structure of these melanins is supposed to be very different [5]. So far, in fungi, three different kinds of melanins have been identified: the most common, the 1,8-dihydroxynaphthalene (DHN) melanin, is a polyketide derivative and its precursor is assembled by a PKS (Figure 1(Ba)); the second one, l-3,4-dihydroxyphenylalanine (l-dopa), is derived by tyrosine degradation and is similar to that produced by mammals (Figure 1(Bb)); finally, pyomelanin was also identified to be produced by some fungi, and is also from tyrosine degradation (Figure 1(Bc)) [6,16]. It is noteworthy that mutants with deleted melanin-related PKSs are normally characterized by the production of colorless spores [17,18,19].

The main studied signaling pathway connected to melanin production is the cAMP signaling pathway [20,21]. However, the deletion of any genes related to the cAMP pathway did not result in total suppression of melanin production [6,22]. This alone suggested that melanin regulation is complex, and likely induced by different signals. Additionally, the involvement of the cAMP pathway during cell wall stress was recently reported in *S. cerevisiae*, revealing a link between the cAMP and the CWI signals [23].

A direct connection between the CWI pathway and melanin production has not been shown thus far, but much experimental evidence has highlighted that this signaling pathway affects melanin production. For example, in *Aspergillus fumigatus*, the deletion of the central CWI MAP kinase, *mpkA*, strongly reduced the expression of melanin-related genes [11,24]. Additionally, the MAD box transcription factor RlmA, the function of which is related to cell wall biosynthesis in different fungi, specifically recognizes a DNA binding motif present in the promoter region of the PKS responsible for DHN-melanin production (named as *pksP*) [25]. Furthermore, in *A. fumigatus*, RlmA affects MpkA activation during cell wall stress, mirroring a conserved role already observed in *S. cerevisiae* [26,27].

Besides *A. fumigatus*, decreased melanin production was observed in other mutants affected in the CWI pathway. In the plant pathogen *Cochliobolus heterostrophus*, deletion of *mps1*, a *mpk1* orthologue, strongly inhibited pigmentation by reducing the expression of DHN-melanin-biosynthetic genes [28]. Similar results were also observed in other plant-pathogenic fungi such as *Botrytis cinerea*, *Alternaria alternata*, *Cryphonectria parasitica*, and even in the mutualistic endophyte *Trichoderma virens* [29,30,31,32].

The function of the CWI pathway is also related to tyrosine degradation, which is the first step for l-dopa melanin biosynthesis. In the human pathogen *Cryptococcus neoformans*, deletion of the *mkk2* gene results in the decrease of melanin formation, and a similar phenotype was observed in *Cryptococcus gattii* after deletion of *mpk1* [33,34]. In the latter case, the inhibition of the CWI pathway affects the transcription of laccases involved in tyrosine degradation, and also influences capsule formation, which is relevant for successful pathogenesis. However, the effect of the cell wall stress on tyrosine degradation seems to be less conserved among fungi. As reported in the model fungus *Neurospora crassa*, the deletion of *mak-1* (*mpk1* orthologue) increases the level of a tyrosinase precursor promoting, in this case, the formation of l-dopa melanin [35]. Additionally, the *mpkA* deletion in *A. fumigatus* positively affects tyrosine degradation by increasing the formation of homogentisate, which is the known precursor of pyomelanin [36].

All these reported examples cannot clarify how melanin production is connected to the CWI. This is mainly due to the lack of information concerning transcriptional regulators involved in the expression of melanin-biosynthetic genes. For example, the deletion of the transcription factor Cmr1 led to a decrease of melanization in both *C. heterostrophus* and *Colletotrichum lagenarium* [28,37]. However, it is still unknown if this factor interacts with any of the promoter regions of melanin-related genes. Two other global regulators involved in DHN-melanin production in *A. fumigatus* were recently reported: the previously mentioned MADS-box RlmA and the basic-helix-loop-helix (bHLH) transcription factor DevR [25]. Independent and combined deletion of the two genes coding for these transcription factors inhibited sporulation and melanization. In *A. fumigatus*, both of these transcription factors cooperatively regulate melanin-biosynthetic genes, and they both specifically bind to the *pksP* promoter region. Targeted DNA mutagenesis of the *pksP* promoter demonstrated that both RlmA and DevR have dual activity depending on the recognized DNA binding domain. In particular, DevR recognizes three different DNA motifs of the *pksP* promoter; the one closer to the ATG start codon, when occupied, represses DHN-melanin expression. However, while the involvement of the CWI pathway in DHN-melanin production is implied, because of the interaction between MpkA and RlmA, the signal regulating DevR is still unknown.

## 4. Mycotoxins, Antibiotics, and Virulence Determinants Affected by the CWI Signaling

Cell wall impairment affects not only the production of fungal melanins, but also the production of many other different secondary metabolites. The change in the metabolism of cell-wall-impaired mutants is quite expected because of changes in the cell shape and misbalance of nutrient acquisition. Moreover, the deletion of *mpk1* in *S. cerevisiae* affects *S*-adenyl-methionine and *S*-adenyl-homocysteine metabolism, which are both important substrates and products in DNA and histone methylation [38]. Histone acetylation, methylation, and phosphorylation potentially play a relevant role in chromatin rearrangements, influencing the ability of transcriptional regulators to access DNA [39]. In filamentous fungi, histone modification was associated with gene cluster activation and secondary metabolite production [40]. Thus, it is conceivable that the CWI signaling pathway also plays a role in the production of secondary metabolites.

Among the many secondary metabolites produced by fungi, mycotoxins are relevant in increasing the environmental competition and in successful pathogenesis for the fungus. *A. fumigatus* produces gliotoxin, a molecule belonging to the family of epidithiodioxopiperazines, which has also been isolated from different fungal species (Figure 1(Bd)) [41]. Gliotoxin is able to kill the social amoeba *Dictyostelium discoideum*, but its relevance during *A. fumigatus* infection is still debatable [42,43]. However, gliotoxin was detected in the lungs of mice and humans infected with *A. fumigatus*, and it is able to inhibit phagocytosis in vitro, meaning that this compound plays a role during infection [44]. Impaired production of gliotoxin was detected in different *A. fumigatus* mutants, such as phosphatase and G couple receptor mutant strains [45,46]. Oppositely, the deletion of the F-box domain protein Fbx15, which is indispensable for oxidative stress response and virulence, determined an increase of gliotoxin production [47]. Concerning the CWI signaling pathway, the deletion of the *mpkA* gene in *A. fumigatus* almost suppresses gliotoxin production, suggesting that this molecule can be also produced in response to cell wall stress [48].

Plant-pathogenic fungi also produce various mycotoxins. It was widely reported that the fungus *A. alternata* produces the ACT toxin (*Alternaria citri* tangerine patho-type), a polyketide derivative particularly harmful for grapefruits and tangerines (Figure 1(Be)) [49]. The deletion of the *Aaslt2* gene not only reduces the *A. alternata* virulence on the tested citruses, but also affects ACT toxin production. These results suggested that, during infection, the cell wall of this plant pathogen is challenged, and the activation of the signaling pathway is important for both invasion and toxin production [30].

The effect of cell wall impairment on plant pathogens’ secondary metabolism has been broadly studied also in *Fusarium* sp. The phyto-pathogenic fungus *Fusarium oxysporum* produces two relevant mycotoxins: beauvericin and fusaric acid [50]. Beauvericin is a cyclohexadepsipeptide with insecticidal activity, also able to induce apoptosis in human cells, while fusaric acid is a mycotoxin with high phytotoxic properties (Figure 1(Bf,g)) [51,52]. Both of the gene clusters responsible for the biosynthesis of these toxins are strongly repressed after the deletion of the *mpk1* orthologue, suggesting that the production of these metabolites can be induced in response to cell wall stress.

Mycotoxins are thought to be used as arming molecules during pathogenic processes, but can also be employed as feeding deterrents. Consequently, mycotoxins are a relevant problem for food production and storage, and understanding their regulation can be used to decrease food contamination levels. A group of infamous mycotoxins produced by *Fusarium* sp. are fumonisins. Fumonisins belong to the family of sphingolipid inhibitors, and they inhibit the activity of the ceramide synthase responsible for the production of phytoceramides, which are sphongolipid precursors in eukaryotic cells [53]. Fumonisins are ranked among the most dangerous known toxins, and their incidence of contamination of post-harvest grain food ranges from 39% (in Europe) to 95% (in North and South America) worldwide [54]. The plant pathogen *Fusarium verticilloides* produces fumonisin B1, which is the most dangerous toxin among the fumonisins (Figure 1(Bh)) [55]. The expression of the fumonsin biosynthetic genes is affected by different stimuli [56]. However, a recent work reported that the deletion of the *bck1* gene, the MAPKKK in the CWI MAP signaling, strongly decreases fumonisin B1 accumulation [57], and similar results were also obtained in the same species by deleting the *mpk1* orthologue *vmk1* [58].

The activation of the CWI pathway was also observed in response to insect grazing. A gene expression study aimed at investigating the effects of *Drosophila melanogaster* on *Aspergillus nidulans* highlighted that the expression of different signaling pathway genes increased during grazing, and this also included *mpkA* [59]. The same group also reported that the soil arthropod *Falsomia candida* induces the production of austinoids and emericellamides after grazing on *A. nidulans* [60]. Emericellamides are mixed cyclic polyketide nonribosomal peptides that exhibit antibacterial activity [61]. On the other hand, austinoids are meroterpenoids with selective species-specific insecticidal activity [62]. We still do not know if these compounds are regulated by CWI signaling, but this data suggests that grazing also induces cell wall stress, and the production of these molecules can be a specific response caused by the interaction and physical contact between insects and fungi.

Besides emericellamides, *A. nidulans* produces the β-lactam antibiotic penicillin, one of the most important antibiotics discovered so far (Figure 1(Bi)). By using RNA-interference, the expression of the gene coding for the protein kinase C PkcA (Pkc1 orthologue), acting upstream of the MAPK module, was silenced. The silencing of the CWI signaling partially inhibits the nuclear localization of the bHLH transcription factor AnBH1, which is involved in penicillin regulation. Consequently, the penicillin titer was strongly reduced during the inhibition of the CWI signaling [63].

After mycotoxins and antibacterial compounds, the CWI signaling was also related to the production of siderophores, which are molecules considered as virulence determinants in many fungi [64]. The ∆*mpkA* mutant in *A. fumigatus* showed an increase of intracellular polyamines, mainly due to the dysregulation of polyamine biosynthetic genes in the mitochondria. Polyamines are important cell components involved in many different cellular processes, such as development, cell transition, and sporulation [65]. Polyamines are derived by l-ornithine, which is also the precursor for the biosynthesis of triacetylfusarinine C, a siderophore produced and secreted by fungi to acquire iron from the environment (Figure 1(Bj)) [2]. Indeed, coupled to polyamines, the inhibition of *A. fumigatus* CWI signaling promotes siderophore accumulation [48]. In *A. fumigatus*, the production of siderophores is strongly regulated by iron homeostasis, and tightly controlled by two transcriptional regulators—HapX and SreA—that co-regulate iron-dependent genes, including those responsible for the biosynthesis of triacetylfusarinine C [66,67]. However, siderophore accumulation in the ∆*mpkA* mutant is not connected to the main iron-regulating complex, signifying that, during cell wall stress, siderophore production occurs in a HapX-/SreA-independent manner [48]. This phenotype was also observed in *F. oxysporum*, suggesting a quite conserved mechanism in filamentous fungi [50].

The effects of CWI signaling on secondary metabolite production were also reported in basidiomycete fungi. The basidiomycete mushroom *Ganoderma lucidum* is a plant pathogen producing many pharmacologically active secondary metabolites. *G. lucidum* produces the meroterpenoid ganoderic acid (Figure 1(Bk)), which can be used as a cholesterol reducing compound [68]. The suppression of CWI signaling, by deleting the *Glslt2* gene, provoked a strong reduction of lanosterol and squalene, which are important components of the cell membrane, but also affected the global production of ganoderic acid [69].

## 5. Perspectives

In the last several years, scientists have been very inventive in developing growth conditions that could activate silent gene clusters. In particular, they have tried to reproduce environmental stress situations to stimulate stress responses and obtain potentially active molecules. For example, fungi have been co-cultivated with different organisms to induce the expression of potential active molecules [70].

The rational manipulation of signaling pathways can be a very straightforward strategy to activate silent gene clusters. However, the works reported so far were mainly focused on investigating the effects of gene deletion/disruption on secondary metabolite production. As shown in this review, in many cases, the inhibition of the CWI signaling pathway negatively affected the production of relevant molecules such as antibiotics and mycotoxins. The discovery of new mycotoxins can be very important because these molecules can also be employed as therapeutics. For example, gliotoxin was found to induce apoptosis in myeloma cell lines, proposing its possible use as a chemotherapeutic drug [41]. Moreover, sphingolipid inhibitors, such as fumonisins, can be potentially used to cure sphingolipid disorders in humans; these disorders have been associated with various diseases, including diabetes and schizophrenia [71]. Following this principle, we are still lacking studies on secondary metabolite cluster expression occurring as a consequence of signaling pathway activation. With the advent of synthetic biology, we are learning daily how to manipulate signaling on demand. The majority of these studies, as usual, have been conducted on model organisms such as *S. cerevisiae* [72]. However, because the signaling pathways are much conserved, it is possible that this knowledge can be transferred to secondary-metabolite-producing fungi in order to discover hidden compounds. A synthetic activation of CWI signaling in *S. cerevisiae* has already been performed by mutating the *pkc1* gene. This change of the arginine in position 398 with an alanine residue induces a constitutively higher activation of the CWI signaling [73]. Similarly, the same mutation in *A. nidulans* was also able to increase the MpkA phosphorylation status [74].

Taken together, all the data presented so far suggests that the manipulation of the CWI signaling pathway could be a useful tool to activate defense mechanisms in fungi, including the production of still-unknown active molecules that could have clinical and industrial applications.

## References

[1] Brakhage A.A. (2013). Regulation of Fungal Secondary Metabolism. Nat. Rev. Microbiol..

[2] Haas H. (2014). Fungal Siderophore Metabolism with a Focus on *Aspergillus fumigatus*. Nat. Prod. Rep..

[3] Hertweck C. (2009). The Biosynthetic Logic of Polyketide Diversity. Angew. Chem. Int. Ed. Engl..

[4] Macheleidt J., Mattern D.J., Fischer J., Netzker T., Weber J., Schroeckh V., Valiante V., Brakhage A.A. (2016). Regulation and Role of Fungal Secondary Metabolites. Annu. Rev. Genet..

[5] Nosanchuk J.D., Stark R.E., Casadevall A. (2015). Fungal Melanin: What Do We Know About Structure?. Front. Microbiol..

[6] Heinekamp T., Thywissen A., Macheleidt J., Keller S., Valiante V., Brakhage A.A. (2012). *Aspergillus fumigatus* Melanins: Interference with the Host Endocytosis Pathway and Impact on Virulence. Front. Microbiol..

[7] Gonzalez A., Hall M.N. (2017). Nutrient Sensing and TOR Signaling in Yeast and Mammals. EMBO J..

[8] Rispail N., Soanes D.M., Ant C., Czajkowski R., Grunler A., Huguet R., Perez-Nadales E., Poli A., Sartorel E., Valiante V. (2009). Comparative Genomics of Map Kinase and Calcium-Calcineurin Signalling Components in Plant and Human Pathogenic Fungi. Fungal Genet. Biol..

[9] Turra D., Segorbe D., di Pietro A. (2014). Protein Kinases in Plant-Pathogenic Fungi: Conserved Regulators of Infection. Annu. Rev. Phytopathol..

[10] Bruder Nascimento A.C., Reis T.F.D., de Castro P.A., Hori J.I., Bom V.L., de Assis L.J., Ramalho L.N., Rocha M.C., Malavazi I., Brown N.A. (2016). Mitogen Activated Protein Kinases Saka (Hog1) and Mpkc Collaborate for *Aspergillus fumigatus* Virulence. Mol. Microbiol..

[11] Altwasser R., Baldin C., Weber J., Guthke R., Kniemeyer O., Brakhage A.A., Linde J., Valiante V. (2015). Network Modeling Reveals Cross Talk of Map Kinases during Adaptation to Caspofungin Stress in *Aspergillus fumigatus*. PLoS ONE.

[12] Saito H. (2010). Regulation of Cross-Talk in Yeast Mapk Signaling Pathways. Curr. Opin. Microbiol..

[13] Torres L., Martin H., Garcia-Saez M.I., Arroyo J., Molina M., Sanchez M., Nombela C. (1991). A Protein Kinase Gene Complements the Lytic Phenotype of *Saccharomyces cerevisiae* Lyt2 Mutants. Mol. Microbiol..

[14] Levin D.E. (2011). Regulation of Cell Wall Biogenesis in *Saccharomyces cerevisiae*: The Cell Wall Integrity Signaling Pathway. Genetics.

[15] Hamel L.P., Nicole M.C., Duplessis S., Ellis B.E. (2012). Mitogen-Activated Protein Kinase Signaling in Plant-Interacting Fungi: Distinct Messages from Conserved Messengers. Plant Cell.

[16] Eisenman H.C., Casadevall A. (2012). Synthesis and Assembly of Fungal Melanin. Appl. Microbiol. Biotechnol..

[17] Langfelder K., Jahn B., Gehringer H., Schmidt A., Wanner G., Brakhage A.A. (1998). Identification of a Polyketide Synthase Gene (Pksp) of *Aspergillus fumigatus* Involved in Conidial Pigment Biosynthesis and Virulence. Med. Microbiol. Immunol..

[18] Akamatsu H.O., Chilvers M.I., Stewart J.E., Peever T.L. (2010). Identification and Function of a Polyketide Synthase Gene Responsible for 1,8-Dihydroxynaphthalene-Melanin Pigment Biosynthesis in Ascochyta Rabiei. Curr. Genet..

[19] Schumacher J. (2016). Dhn Melanin Biosynthesis in the Plant Pathogenic Fungus *Botrytis cinerea* Is Based on Two Developmentally Regulated Key Enzyme (Pks)-Encoding Genes. Mol. Microbiol..

[20] Brakhage A.A., Liebmann B. (2005). *Aspergillus fumigatus* Conidial Pigment and Camp Signal Transduction: Significance for Virulence. Med. Mycol..

[21] Alspaugh J.A., Perfect J.R., Heitman J. (1997). *Cryptococcus neoformans* Mating and Virulence Are Regulated by the G-Protein Alpha Subunit Gpa1 and Camp. Genes Dev..

[22] Calvo A.M., Wilson R.A., Bok J.W., Keller N.P. (2002). Relationship between Secondary Metabolism and Fungal Development. Microbiol. Mol. Biol. Rev..

[23] Garcia R., Bravo E., Diez-Muniz S., Nombela C., Rodriguez-Pena J.M., Arroyo J. (2017). A Novel Connection between the Cell Wall Integrity and the Pka Pathways Regulates Cell Wall Stress Response in Yeast. Sci. Rep..

[24] Muller S., Baldin C., Groth M., Guthke R., Kniemeyer O., Brakhage A.A., Valiante V. (2012). Comparison of Transcriptome Technologies in the Pathogenic Fungus *Aspergillus fumigatus* Reveals Novel Insights into the Genome and Mpka Dependent Gene Expression. BMC Genom..

[25] Valiante V., Baldin C., Hortschansky P., Jain R., Thywissen A., Strassburger M., Shelest E., Heinekamp T., Brakhage A.A. (2016). The *Aspergillus fumigatus* Conidial Melanin Production Is Regulated by the Bifunctional Bhlh Devr and Mads-Box Rlma Transcription Factors. Mol. Microbiol..

[26] Rocha M.C., Fabri J.H., de Godoy K.F., de Castro P.A., Hori J.I., da Cunha A.F., Arentshorst M., Ram A.F., van den Hondel C.A., Goldman G.H. (2016). *Aspergillus fumigatus* Mads-Box Transcription Factor Rlma Is Required for Regulation of the Cell Wall Integrity and Virulence. G3 Genes Genomes Genet..

[27] Dodou E., Treisman R. (1997). The *Saccharomyces cerevisiae* Mads-Box Transcription Factor Rlm1 Is a Target for the Mpk1 Mitogen-Activated Protein Kinase Pathway. Mol. Cell. Biol..

[28] Eliahu N., Igbaria A., Rose M.S., Horwitz B.A., Lev S. (2007). Melanin Biosynthesis in the Maize Pathogen *Cochliobolus heterostrophus* Depends on Two Mitogen-Activated Protein Kinases, Chk1 and Mps1, and the Transcription Factor Cmr1. Eukaryot. Cell.

[29] So K.K., Ko Y.H., Chun J., Kim J.M., Kim D.H. (2017). Mutation of the Slt2 Ortholog from *Cryphonectria parasitica* Results in Abnormal Cell Wall Integrity and Sectorization with Impaired Pathogenicity. Sci. Rep..

[30] Yago J.I., Lin C.H., Chung K.R. (2011). The Slt2 Mitogen-Activated Protein Kinase-Mediated Signalling Pathway Governs Conidiation, Morphogenesis, Fungal Virulence and Production of Toxin and Melanin in the Tangerine Pathotype of *Alternaria alternata*. Mol. Plant Pathol..

[31] Kumar A., Scher K., Mukherjee M., Pardovitz-Kedmi E., Sible G.V., Singh U.S., Kale S.P., Mukherjee P.K., Horwitz B.A. (2010). Overlapping and Distinct Functions of Two *Trichoderma virens* Map Kinases in Cell-Wall Integrity, Antagonistic Properties and Repression of Conidiation. Biochem. Biophys. Res. Commun..

[32] Liu W., Soulie M.C., Perrino C., Fillinger S. (2011). The Osmosensing Signal Transduction Pathway from *Botrytis cinerea* Regulates Cell Wall Integrity and Map Kinase Pathways Control Melanin Biosynthesis with Influence of Light. Fungal Genet. Biol..

[33] Gerik K.J., Donlin M.J., Soto C.E., Banks A.M., Banks I.R., Maligie M.A., Selitrennikoff C.P., Lodge J.K. (2005). Cell Wall Integrity Is Dependent on the Pkc1 Signal Transduction Pathway in *Cryptococcus neoformans*. Mol. Microbiol..

[34] Ngamskulrungroj P., Price J., Sorrell T., Perfect J.R., Meyer W. (2011). *Cryptococcus gattii* Virulence Composite: Candidate Genes Revealed by Microarray Analysis of High and Less Virulent Vancouver Island Outbreak Strains. PLoS ONE.

[35] Park G., Pan S., Borkovich K.A. (2008). Mitogen-Activated Protein Kinase Cascade Required for Regulation of Development and Secondary Metabolism in *Neurospora crassa*. Eukaryot. Cell.

[36] Valiante V., Jain R., Heinekamp T., Brakhage A.A. (2009). The Mpka Map Kinase Module Regulates Cell Wall Integrity Signaling and Pyomelanin Formation in *Aspergillus fumigatus*. Fungal Genet. Biol..

[37] Tsuji G., Kenmochi Y., Takano Y., Sweigard J., Farrall L., Furusawa I., Horino O., Kubo Y. (2000). Novel Fungal Transcriptional Activators, Cmr1p of *Colletotrichum lagenarium* and Pig1p of Magnaporthe Grisea, Contain Cys2his2 Zinc Finger and Zn(Ii)2cys6 Binuclear Cluster DNA-Binding Motifs and Regulate Transcription of Melanin Biosynthesis Genes in a Developmentally Specific Manner. Mol. Microbiol..

[38] Breunig J.S., Hackett S.R., Rabinowitz J.D., Kruglyak L. (2014). Genetic Basis of Metabolome Variation in Yeast. PLoS Genet..

[39] Bannister A.J., Kouzarides T. (2011). Regulation of Chromatin by Histone Modifications. Cell Res..

[40] Nutzmann H.W., Fischer J., Scherlach K., Hertweck C., Brakhage A.A. (2013). Distinct Amino Acids of Histone H3 Control Secondary Metabolism in *Aspergillus nidulans*. Appl. Environ. Microbiol..

[41] Scharf D.H., Brakhage A.A., Mukherjee P.K. (2016). Gliotoxin—Bane or Boon?. Environ. Microbiol..

[42] Hillmann F., Novohradska S., Mattern D.J., Forberger T., Heinekamp T., Westermann M., Winckler T., Brakhage A.A. (2015). Virulence Determinants of the Human Pathogenic Fungus *Aspergillus fumigatus* Protect against Soil Amoeba Predation. Environ. Microbiol..

[43] Cramer R.A., Gamcsik M.P., Brooking R.M., Najvar L.K., Kirkpatrick W.R., Patterson T.F., Balibar C.J., Graybill J.R., Perfect J.R., Abraham S.N. (2006). Disruption of a Nonribosomal Peptide Synthetase in *Aspergillus fumigatus* Eliminates Gliotoxin Production. Eukaryot. Cell.

[44] Cerqueira L.B., de Francisco T.M., Gasparetto J.C., Campos F.R., Pontarolo R. (2014). Development and Validation of an Hplc-Ms/Ms Method for the Early Diagnosis of Aspergillosis. PLoS ONE.

[45] Winkelstroter L.K., Dolan S.K., Reis T.F.D., Bom V.L., de Castro P.A., Hagiwara D., Alowni R., Jones G.W., Doyle S., Brown N.A. (2015). Systematic Global Analysis of Genes Encoding Protein Phosphatases in *Aspergillus fumigatus*. G3 Genes Genomes Genet..

[46] Jung M.G., Kim S.S., Yu J.H., Shin K.S. (2016). Characterization of Gprk Encoding a Putative Hybrid G-Protein-Coupled Receptor in *Aspergillus fumigatus*. PLoS ONE.

[47] Johnk B., Bayram O., Abelmann A., Heinekamp T., Mattern D.J., Brakhage A.A., Jacobsen I.D., Valerius O., Braus G.H. (2016). Scf Ubiquitin Ligase F-Box Protein Fbx15 Controls Nuclear Co-Repressor Localization, Stress Response and Virulence of the Human Pathogen *Aspergillus fumigatus*. PLoS Pathog..

[48] Jain R., Valiante V., Remme N., Docimo T., Heinekamp T., Hertweck C., Gershenzon J., Haas H., Brakhage A.A. (2011). The Map Kinase Mpka Controls Cell Wall Integrity, Oxidative Stress Response, Gliotoxin Production and Iron Adaptation in *Aspergillus fumigatus*. Mol. Microbiol..

[49] Ito K., Tanaka T., Hatta R., Yamamoto M., Akimitsu K., Tsuge T. (2004). Dissection of the Host Range of the Fungal Plant Pathogen *Alternaria alternata* by Modification of Secondary Metabolism. Mol. Microbiol..

[50] Ding Z., Li M., Sun F., Xi P., Sun L., Zhang L., Jiang Z. (2015). Mitogen-Activated Protein Kinases Are Associated with the Regulation of Physiological Traits and Virulence in *Fusarium oxysporum* f. sp. *Cubense*. PLoS ONE.

[51] Niehaus E.M., von Bargen K.W., Espino J.J., Pfannmuller A., Humpf H.U., Tudzynski B. (2014). Characterization of the Fusaric Acid Gene Cluster in *Fusarium fujikuroi*. Appl. Microbiol. Biotechnol..

[52] Logrieco A., Moretti A., Castella G., Kostecki M., Golinski P., Ritieni A., Chelkowski J. (1998). Beauvericin Production by *Fusarium* Species. Appl. Environ. Microbiol..

[53] Wang E., Norred W.P., Bacon C.W., Riley R.T., Merrill A.H. (1991). Inhibition of Sphingolipid Biosynthesis by Fumonisins. Implications for Diseases Associated with *Fusarium moniliforme*. J. Biol. Chem..

[54] Lee H.J., Ryu D. (2017). Worldwide Occurrence of Mycotoxins in Cereals and Cereal-Derived Food Products: Public Health Perspectives of Their Co-Occurrence. J. Agric. Food Chem..

[55] Shephard G.S., van der Westhuizen L., Sewram V. (2007). Biomarkers of Exposure to Fumonisin Mycotoxins: A Review. Food Addit. Contam..

[56] Rocha L.O., Barroso V.M., Andrade L.J., Pereira G.H., Ferreira-Castro F.L., Duarte A.P., Michelotto M.D., Correa B. (2015). Fum Gene Expression Profile and Fumonisin Production by *Fusarium verticillioides* Inoculated in *Bt* and Non-*Bt* Maize. Front. Microbiol..

[57] Zhang C., Wang J., Tao H., Dang X., Wang Y., Chen M., Zhai Z., Yu W., Xu L., Shim W.B. (2015). Fvbck1, a Component of Cell Wall Integrity Map Kinase Pathway, Is Required for Virulence and Oxidative Stress Response in Sugarcane Pokkah Boeng Pathogen. Front. Microbiol..

[58] Zhang Y., Choi Y.E., Zou X., Xu J.R. (2011). The Fvmk1 Mitogen-Activated Protein Kinase Gene Regulates Conidiation, Pathogenesis, and Fumonisin Production in *Fusarium verticillioides*. Fungal Genet. Biol..

[59] Caballero Ortiz S., Trienens M., Rohlfs M. (2013). Induced Fungal Resistance to Insect Grazing: Reciprocal Fitness Consequences and Fungal Gene Expression in the *Drosophila*-*Aspergillus* Model System. PLoS ONE.

[60] Doll K., Chatterjee S., Scheu S., Karlovsky P., Rohlfs M. (2013). Fungal Metabolic Plasticity and Sexual Development Mediate Induced Resistance to Arthropod Fungivory. Proc. Biol. Sci..

[61] Chiang Y.M., Szewczyk E., Nayak T., Davidson A.D., Sanchez J.F., Lo H.C., Ho W.Y., Simityan H., Kuo E., Praseuth A. (2008). Molecular Genetic Mining of the *Aspergillus* Secondary Metabolome: Discovery of the Emericellamide Biosynthetic Pathway. Chem. Biol..

[62] Valiante V., Mattern D.J., Schuffler A., Horn F., Walther G., Scherlach K., Petzke L., Dickhaut J., Guthke R., Hertweck C. (2017). Discovery of an Extended Austinoid Biosynthetic Pathway in *Aspergillus calidoustus*. ACS Chem. Biol..

[63] Herrmann M., Sprote P., Brakhage A.A. (2006). Protein Kinase C (Pkca) of *Aspergillus nidulans* Is Involved in Penicillin Production. Appl. Environ. Microbiol..

[64] Haas H., Eisendle M., Turgeon B.G. (2008). Siderophores in Fungal Physiology and Virulence. Annu. Rev. Phytopathol..

[65] Jin Y., Bok J.W., Guzman-de-Pena D., Keller N.P. (2002). Requirement of Spermidine for Developmental Transitions in *Aspergillus nidulans*. Mol. Microbiol..

[66] Gsaller F., Hortschansky P., Beattie S.R., Klammer V., Tuppatsch K., Lechner B.E., Rietzschel N., Werner E.R., Vogan A.A., Chung D. (2014). The Janus Transcription Factor Hapx Controls Fungal Adaptation to Both Iron Starvation and Iron Excess. EMBO J..

[67] Schrettl M., Beckmann N., Varga J., Heinekamp T., Jacobsen I.D., Jochl C., Moussa T.A., Wang S., Gsaller F., Blatzer M. (2010). Hapx-Mediated Adaption to Iron Starvation Is Crucial for Virulence of *Aspergillus fumigatus*. PLoS Pathog..

[68] Hajjaj H., Mace C., Roberts M., Niederberger P., Fay L.B. (2005). Effect of 26-Oxygenosterols from *Ganoderma lucidum* and Their Activity as Cholesterol Synthesis Inhibitors. Appl. Environ. Microbiol..

[69] Zhang G., Sun Z., Ren A., Shi L., Shi D., Li X., Zhao M. (2017). The Mitogen-Activated Protein Kinase Glslt2 Regulates Fungal Growth, Fruiting Body Development, Cell Wall Integrity, Oxidative Stress and Ganoderic Acid Biosynthesis in *Ganoderma lucidum*. Fungal Genet. Biol..

[70] Netzker T., Fischer J., Weber J., Mattern D.J., Konig C.C., Valiante V., Schroeckh V., Brakhage A.A. (2015). Microbial Communication Leading to the Activation of Silent Fungal Secondary Metabolite Gene Clusters. Front. Microbiol..

[71] Canals D., Perry D.M., Jenkins R.W., Hannun Y.A. (2011). Drug Targeting of Sphingolipid Metabolism: Sphingomyelinases and Ceramidases. Br. J. Pharmacol..

[72] Furukawa K., Hohmann S. (2013). Synthetic Biology: Lessons from Engineering Yeast Mapk Signalling Pathways. Mol. Microbiol..

[73] Watanabe M., Chen C.Y., Levin D.E. (1994). *Saccharomyces cerevisiae* Pkc1 Encodes a Protein Kinase C (Pkc) Homolog with a Substrate Specificity Similar to That of Mammalian Pkc. J. Biol. Chem..

[74] Katayama T., Ohta A., Horiuchi H. (2015). Protein Kinase C Regulates the Expression of Cell Wall-Related Genes in Rlma-Dependent and Independent Manners in *Aspergillus nidulans*. Biosci. Biotechnol. Biochem..

